# Genipin-crosslinked chitosan/alginate/alumina nanocomposite gels for 3D bioprinting

**DOI:** 10.1007/s00449-021-02650-3

**Published:** 2021-10-18

**Authors:** Jessica Condi Mainardi, Kurosch Rezwan, Michael Maas

**Affiliations:** 1 Keramische Werkstoffe und Bauteile/Advanced Ceramics, Universität Bremen, Am Biologischen Garten 2, IW 3, Raum 2140, 28359 Bremen, Germany; 2 MAPEX Center for Materials and Processes, University of Bremen, Am Fallturm 1, 28359 Bremen, Germany

**Keywords:** 3D bioprinting, Genipin, Chitosan, Alginate, Alumina, Nanocomposite

## Abstract

Immobilizing microorganisms inside 3D printed semi-permeable substrates can be desirable for biotechnological processes since it simplifies product separation and purification, reducing costs, and processing time. To this end, we developed a strategy for synthesizing a feedstock suitable for 3D bioprinting of mechanically rigid and insoluble materials with embedded living bacteria. The processing route is based on a highly particle-filled alumina/chitosan nanocomposite gel which is reinforced by (a) electrostatic interactions with alginate and (b) covalent binding between the chitosan molecules with the mild gelation agent genipin. To analyze network formation and material properties, we characterized the rheological properties and printability of the feedstock gel. Stability measurements showed that the genipin-crosslinked chitosan/alginate/alumina gels did not dissolve in PBS, NaOH, or HCl after 60 days of incubation. Alginate-containing gels also showed less swelling in water than gels without alginate. Furthermore, *E. coli* bacteria were embedded in the nanocomposites and we analyzed the influence of the individual bioink components as well as of the printing process on bacterial viability. Here, the addition of alginate was necessary to maintain the effective viability of the embedded bacteria, while samples without alginate showed no bacterial viability. The experimental results demonstrate the potential of this approach for producing macroscopic bioactive materials with complex 3D geometries as a platform for novel applications in bioprocessing.

## Introduction

Developing a bioink to produce mechanically stable materials with embedded bacteria and with customized porous structure by 3D bioprinting could lead to innovative bioreactor concepts. In such applications, both the support material and the embedded cells have to survive the rigors of long-term continuous flow processing or in related conditions. Likewise, the fabrication of complex porous geometries for bioreactors with highly accessible surface areas necessitates innovative printing strategies that result in rigid and insoluble materials. While bioprinting of cells embedded inside soft hydrogels has been well established especially in the field of regenerative medicine [1], printing of biomaterials that incorporate living cells is still very challenging, especially considering that the production of mechanically rigid and insoluble substrates usually requires non-biocompatible processes, like chemical crosslinking or sintering [2].

Bioinks are defined as “a formulation of cells suitable for processing by an automated biofabrication technology” [3] and to print living cells, both the support material and the crosslinking method should be compatible with cells and result in materials with high mechanical stability and insolubility [4]. Accordingly, bioinks must fulfill two primary criteria: high cell viability and high printability, the latter being the ability to form 3D structures with good fidelity and integrity [2]. Ideally, printable materials should exhibit a solid-like behavior of the printed filaments, which should be strong enough to support the deposition of further layers. Furthermore, printed filaments should stack with each other without merging and gelation should occur after filament extrusion to avoid nozzle blocking [5]. Hence, to achieve a successful 3D bioprinting process, physical and physiological properties need to be carefully tuned to ensure both good printability and high cell viability [6].

In a previous paper we described the immobilization of bacteria *E. coli* and *B. subtilis* into rigid alginate/alumina nanocomposites by gel casting with good mechanical properties and high cell viability after 60 days of storage. Alginate is a gelling polysaccharide with high biocompatibility that undergoes a biocompatible gelation process with Ca^2+^ ions. Forming a highly filled nanocomposite of alginate with alumina results in enhanced mechanical properties of the gel, particularly greatly reduced shrinking during drying and net-near-shape processing during extrusion and molding. This makes this bionanocomposite a good candidate for a feedstock for 3D bioprinting [7]. However, due to the lack of covalent crosslinking in this hydrogel/ceramic nanocomposite, the material degrades over time and is therefore unsuited for long-term application. To further enhance this nanocomposite hydrogel, oppositely charged polymers (e.g., chitosan) can be used to crosslink alginate by forming polyelectrolyte complexes [8–11]. This strategy is widely used for cell encapsulation, usually by first encapsulating cells in alginate microspheres via ionotropic gelation, followed by a coating with chitosan via the principle of polyelectrolyte complexation [12–14]. By coating alginate with chitosan, a slower degradation rate could be demonstrated while high bacteria viability was achieved by protecting bacteria within the alginate from the antibacterial properties of chitosan [15]. Colosi et al. used the same principle for 3D printing by first printing alginate suspensions (without cells) followed by a coating step with chitosan [16], and the coating was further reinforced by covalent crosslinking to ensure the structural stability of the materials in culture media for a prolonged period of time.

Chitosan is a biocompatible product of the deacetylation of chitin, which is found in crustacean shells. The resulting polymer is based on a polysaccharide backbone with a high quantity of primary amine functional groups and it is able to form a hydrogel [17–19]. Chitosan dissolves in acidic pH (i.e., pH < 6.2) but not in neutral pH. The use of chitosan has notably increased during the past years in part owing to the cheap and natural source of this polymer [20, 21], its antimicrobial activity (depending on its molecular weight and acetylation degree) [22, 23] along with its chemical properties. The main application fields for this polymer are in the food industry as emulsifier and feed additives [24], as well as in the pharmaceutical industry as encapsulation of agents for drug delivery [25], in the packaging industry [26], in tissue engineering [27, 28], and in bioprocessing [22, 29].

To date, 3D bioprinting of chitosan corresponds to just approx. 4% of bioprinting publications but has shown promising results in the field of tissue engineering. In the field of bioprocessing, the use of chitosan in conjunction with 3D printing is still nascent [23, 30]. However, chitosan gels have low mechanical resistance, which is one of the main limitations of their use in 3D bioprinting. To overcome this drawback, chitosan is often used in combination with other components to enhance its mechanical properties [18]. Several reinforcement strategies can be used, such as admixing of nanoparticles, ionotropic gelation [31–33], polyelectrolyte complexation, and a variety of chemical reactions for covalent crosslinking [17, 34, 35]. Integrating nanoparticles into the chitosan gel to form a composite can significantly enhance mechanical resistance [36] and printability [37] and also add extra functionality to the gel, such as conductivity [37], fluorescence [38], or antibacterial properties [39]. For example, Maturavongsadit et al. [40] developed a bioink based on a thermogelling chitosan, glycerophosphate, hydroxyethyl cellulose, and cellulose nanocrystals containing pre-osteoblast cells (MC3T3-E1) for bone tissue engineering. The addition of cellulose nanocrystals into the bioink resulted in a 20% increment on both viscosity and yield stress, as well as nanocrystals promoted a greater osteogenesis of the cells in chitosan scaffolds by higher calcium mineralization and extracellular matrix formation. Moreover, based on its abundance of amine groups, chitosan is often covalently crosslinked with carboxyl-rich polymers, like alginate via carbodiimide chemistry [41, 42] or with glutaraldehyde [43]. However, these reactions are not biocompatible and would therefore necessitate cell immobilization subsequent to chemical processing.

An innovative alternative for covalently crosslinking chitosan is to use genipin as crosslinking agent, which is a natural molecule extracted from the fruits of *Gardenia jasminoides* [44]. Genipin is a small molecule with very low toxicity and it is able to crosslink proteins and polysaccharides containing residues with amine groups [21, 45, 46]. This crosslinking proceeds in two separate reactions, first the formation of a heterocyclic compound of genipin linked to the glucosamine residue in chitosan and second a nucleophilic substitution of its ester group to form a secondary amide link with another chitosan molecule [47–49]. Simultaneously, polymerization can take place between genipin molecules already linked to amino groups of chitosan, which leads to the crosslinking of amino groups by short genipin copolymers. For example, Hafezi et al. [50] developed a bioink with keratinocyte and human dermal fibroblast cells based on chitosan and crosslinked with genipin. Cell viability was still 85% seven days after the printing process, crosslinking, and incubation. Furthermore, morphological studies showed that the cells remained mobile in the constructs after crosslinking. However, the genipin–chitosan crosslinking reaction takes several hours [51, 52] and is therefore not fast enough to immediately reinforce 3D printed structures that otherwise would not maintain their shape after printing [50]. Therefore, a combination of reinforcement strategies might be required to improve printability, shape fidelity, and long-term stability [37, 53–55].

In this paper, we report the development of a feedstock to increment accessible surface via 3D bioprinting which utilizes the crosslinking reaction between chitosan and genipin. To achieve printability and structural fidelity, the feedstock is based on a highly filled alumina/chitosan nanocomposite gel which is combined with different admixtures of alginate to tailor the rheological properties of this gel and to enhance its compatibility with embedded bacteria. The slow crosslinking reaction between genipin and chitosan enables the addition of genipin before printing without blocking the printing nozzle. Furthermore, the covalent crosslinking reaction should prevent long-term dissolution of the samples. The rheological properties of the feedstock were analyzed in depth providing information on network structure and printability. Detailed feedstock printability characterization and assessment of long-term stability were carried out via image analysis of printed constructs. Living *Escherichia coli* were integrated into the feedstock to test the compatibility of the nanocomposite with a model bacteria to ensure comparability with the literature and to demonstrate potential applications in bioprocessing. Therefore, bacterial viability was analyzed as a function of the different material components as well as of the printing process.

## Materials and methods

### Chemicals

Chitosan with a deacetylation degree of 85 ± 5%, *η* = 15–25 cps. Alumina powder (CT 3000 SG, d50 = 500 nm, purity 99.78%) was purchased from Almatis (Ludwigshafen am Rhein, Germany). Alginic acid sodium salt from brown algae—medium viscosity (Product Number.: A2033), glucose (product Number: G8270), phosphate buffered saline (PBS) (Product Number: P4417), lysogeny broth (LB) medium (product Number: L3022), glutaraldehyde solution (product number: G5882), and sodium chloride (product number: S7653) were purchased from Sigma-Aldrich Chemie GmbH (Munich, Germany). Genipin (product number: 6902-77-8) was purchased from Challenge Bioproducts Co., Ltd. (Douliu city, Taiwan). Three different bacterial viability assays were used in this work: BacTiter-Glo (product number: G8231) from Promega (Walldorf, Germany), an assay based on resazurin salt (product number: Cay14322) was purchased from Cayman chemical (Hamburg, Germany), and WST-1 assay (product number: 5015944001) was obtained from Roche (Mannheim, Germany).

### Bacteria strain and culture conditions

The bacterial strain *Escherichia coli* K12 (DMS 1077) was obtained from Leibniz Institute DSMZ (Braunschweig, Germany), and the bacterial culture was set to grow overnight in sterile LB medium at 37 °C under agitation at 150 rpm in an incubator (Heidolph Unimax 1010, Schwalbach, Germany). Thereafter, the cell suspension was centrifuged at 2500 rpm for 10 min to obtain a cell pellet. Then, the supernatant was discarded, and the bacteria pellet was resuspended with PBS until the desired concentration, by adjusting optical density at 595 nm, to approx. 32.5 × 10^8^ cfu/mL of *E. coli*.

### Feedstock preparation

All dispersions and solutions were prepared under sterile conditions. First, 1.3 g chitosan was dissolved in 50 mL of 0.1% acetic acid solution at room temperature (RT) via a dispermat (IKA RW20.n—Staufen, Germany) for 30 min at 600 rpm. After total dissolution of chitosan, alumina powder was slowly added into the solution and was further stirred at 1200 rpm for 30 min for homogenization. Thereafter, the pH was adjusted to 6 with 1 M NaOH solution. In parallel, 1.3 g alginate was dissolved in 50 mL millipore water at room temperature (RT) using a dispermat (IKA RW20.n—Staufen, Germany) for 30 min at 600 rpm, and thereafter, NaCl was added to a final concentration of 0.7 wt. % and mixed until dissolution. Then, 20 mL of the 2.5 wt.% alginate solution was added into the chitosan/alumina suspension to a total polymer concentration of 2.5% which contains 30 wt.% of alginate and stirred for homogenization at 1000 rpm for 20 min. For all suspensions, the ceramic and overall polymer content was maintained constant at 42 vol.% and 2.5 wt.%, respectively. A solution without alginate was also produced for comparison purposes (see Table 1). After that, the suspension could be further processed or stored at 4 °C.

**Table 1:** Final concentrations of the feedstock composition

Sample	Chitosan (mg/mL)	Alumina (g/mL)	Alginate (mg/mL)	Genipin (mM)
0%	13.5	1.23	0	0.26
30%	9.45	1.23	4.05	0.20

### Bionanocomposite production

**Fig. 1:**
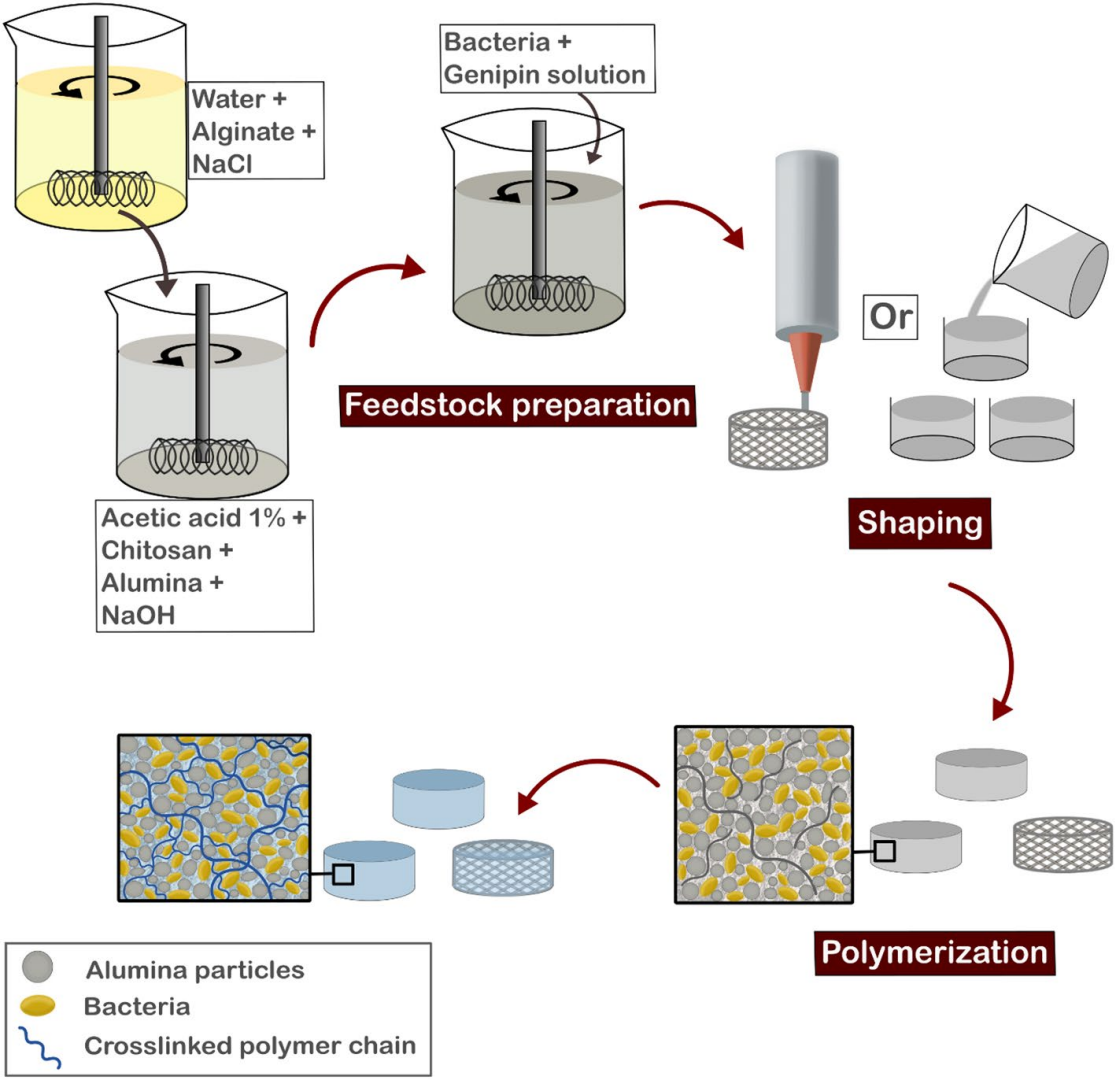
Scheme illustrating the bionanocomposite processing route. First chitosan is dissolved in water followed by the addition of alumina powder. After homogenization of the mixture, alginate solution and thereafter microorganisms can be incorporated into the suspension. Lastly, genipin is added as a crosslinking agent. The feedstock can be shaped by either gel casting or 3D printing

After removal from storage at 4 °C, the suspensions were stirred for five minutes at 1200 rpm in sterile conditions to increase the suspensions temperature to 25 °C, followed by the addition of 2 mL of LB medium (Fig. 1). After homogenization, the stirring velocity was decreased to 400 rpm and the bacteria suspension in PBS was added to the mixture, followed by intense stirring at 1000 rpm for 30 s. A 4 wt.% genipin stock solution was prepared by dissolving genipin in absolute ethanol which was ultrasonicated for 5 min. Then, millipore water was added to decrease the ethanol concentration to 20 vol.% and obtain the final genipin concentration of 4 wt.%. Then, the genipin solution was added to the feedstock to a final concentration of 0.44 wt.% relative to chitosan weight, resulting in a solution with a final concentration of 0.26 or 0.20 mM of genipin, for 0% and 30% alginate formulations, respectively. A summary of all concentrations used for material production is shown in Table 1. It is important to note that the overall polymer concentration (chitosan + alginate) as well as the overall concentration of ceramic particles was maintained constant for comparison purposes. Furthermore, the concentration of genipin was maintained constant in regard to the chitosan concentration, since genipin crosslinking just occurs with chitosan. After genipin addition, the feedstock was mixed at 1200 rpm for 30 s. Subsequently, the feedstock was shaped by two different processing routes: gel casting or 3D printing. For the gel-cast samples, the bioink was cast into small petri dishes (⌀ 35 mm) at room temperature, which were partially covered with Parafilm to avoid significant drying. 3D printed samples were printed into lattice cubes (2 × 2 × 1 cm) using the printer Inkredible (Cellink, Gothenburg, Sweden) in a 6-well plate with a conical precision tip nozzle (⌀ 940 μm) with an extrusion air pressure of 20 ± 5 kPa, a printing speed of 10 mm/s, and a printing temperature of 30 °C—similar as other publications using chitosan-based bioink [40, 50]. The numerical controlled programming language G-code with the printing commands was generated using the Cellink HeartWare 2.4.1 software, from Cellink, with a 67% infill density and 0.85 mm layer high. Afterward, each well was filled with PBS to avoid drying. Additionally, some wells were filled with a 6.6% LB solution. Then, the shaped samples (gel-cast and 3D printed samples) were stored in an incubator at 37 °C for 24 h without shaking for crosslinking. Thereafter, gel-cast and 3D printed samples were removed from the incubator and washed with PBS to remove any freely suspended bacteria in the supernatant before further characterization.

### Rheological characterization

Rheological characterization of the different materials was carried out in a stress-controlled rotational rheometer, Kinexus pro (Malvern Panalytical, Kassel, Germany). Rheology tests were carried out by depositing the gels between the rheometer base plate and a ⌀ 20 mm parallel plate geometry at a gap of 0.5 mm and a temperature of 37 °C. All experiments were performed three times to assure reproducibility and using a solvent trap to avoid drying. Additionally, the yield point of the suspensions was determined with a continuous shear rate ramp from 0.001 to 100 s^−1^. The shear viscosity was acquired using a stepped shear rate test at which each shear rate was held for one minute to allow for equilibration at each shear rate, while the yield point was determined using a shear rate ramp at which the shear rated is increased continuously. Therefore, the first is a more steady-state test to understand how the feedstock acts during the printing process and the second is a dynamic test to simulate the flow initiation. The shear rate ramp is a standard method to characterize a feedstock’s yield point, while the stepped shear rate test is used to characterize fluid behavior, such as Newtonian, shear thinning, or shear thickening behavior [4]. Furthermore, shear rates inside the nozzle were calculated based on Blaeser et al. [56] and Balani et al. [57] and for both cases shear rates lower than 1000 s^−1^ were obtained. Thus, stepped shear rate increment experiments were performed to analyze the change of viscosity with a shear rate range between 0.01 and 1000 s^−1^ with 8 logarithmic increments per decade of shear rate and 1 min holding time at each shear rate. Thixotropic behavior was analyzed by a three-step shear test with a shear rate of 0.05 s^−1^ and a holding time of 60 s for the first step, 50 s^−1^ for 60 s for the second step, and 0.05 s^−1^ for 60 s for the third step. Oscillatory tests were performed to analyze the viscoelastic behavior of the gels before and after genipin crosslinking. First, amplitude tests were performed between 0.01 and 100% amplitude with a constant frequency of 0.5 Hz to determine the linear viscoelastic (LVE) range or rather regions of parallel moduli which indicate reversible deformation. Then, time tests were performed at a constant frequency of 0.5 Hz, at the amplitudes of the respective LVE regions (0.05% and 10%), and over a duration of 8 h.

### Feedstock printability characterization

Feedstock printability, namely, the ability to form 3D structure with good fidelity and integrity, was evaluated by means of image analysis. Ideally, printed constructs should display a clear morphology with smooth surfaces, constant diameters after printing, and the ability to stack with other filaments without merging. Therefore, for regular lattice grid structures, square-shaped holes should in principle be formed in the interstitial spaces between interconnected filaments in the fabricated constructs. Ouyang et. al. [58] proposed an approach to define bioink printability (Pr) based on the analysis of the hole shape using the following function:

$$\Pr = ~\frac{{L^{2} }}{{16A}},$$

where *L* means hole perimeter and *A* means hole area. Ideally, Pr values should be 1 so that the interconnected channels of the constructs would form a square shape. Pr > 1 means that the feedstock shows a high solid-like behavior, usually due to an early crosslinking, and printing constructs show a fractured morphology with irregular filaments, while Pr < 1 means insufficient crosslinking, where the feedstock shows a liquid-like behavior and filaments merge with each other, forming circular holes rather than square holes. To determine Pr values of printed samples, the spacing between interconnected channels was analyzed after printing 3 and 8 layers with a digital optical microscope (VHX-5000 from Keyence—Neu-Isenburg, Germany) after drying at ambient conditions. Perimeter and area of the space between the interconnected channels were determined using microscope’s software VHX-5000 from Keyence.

### Material dissolution stability

Chitosan/alumina nanocomposite gels with and without alginate were prepared and incubated for crosslinking with genipin for 24 h at 37 °C. Thereafter, the gel-cast (non-printed) samples were cut into rectangular pieces (3 × 1 × 0.3 cm) and deformed in different directions for visualization. Furthermore, for testing the long-term stability in various media, 2.5 g of chitosan/alumina composites without genipin crosslinking was analyzed, while genipin-crosslinked chitosan/alumina composites with and without alginate were cut into cuboid geometries of 1.5 × 1 × 0.5 cm size and submerged in four different media: water, PBS, 1 M NaOH, and 1 M HCl. Samples were rigorously hand shaken for 10 s every four days and deviations in size or shape were visually analyzed after 1 and 60 days.

### Bacterial viability test

The influence of genipin on the viability of *E. coli* was measured by incubating suspended bacteria in PBS with different concentrations of genipin: 1 mM, 0.75 mM, 0.5 mM, 0.25 mM, and a control of cells just in PBS (0 mM). Bacteria-containing suspensions were then incubated at 37 °C and 150 rpm and bacterial viability was determined after 5 and 24 h incubation using BacTiter-Glo assay and measuring the luminescence with Chameleon V plate reader, from Hidex (Mainz, Germany).

The effective viability of accessible immobilized bacteria was measured with an assay containing resazurin sodium salt. Viable cells with active metabolism can reduce resazurin into resorufin, which is pink and fluorescent. The product can be quantified by measuring the fluorescence signal using a photometer. For that, three replicates of both gel-cast and 3D printed samples with and without alginate containing *E. coli* were first incubated for 24 h for genipin crosslinking. Thereafter, samples were washed with PBS and positioned in a 6-well plate, which was afterward filled with a solution of PBS with 10% of resazurin stock solution, and were incubated protected from light for further 4 h at 37 °C and 160 rpm. Resazurin stock solutions were produced as follows: 1 g of resazurin salt was dissolved in 100 mL of sterile PBS and stirred for homogenization, followed by a filtration step with 0.2 µm filters under sterile conditions to obtain resazurin stock solutions with a concentration of 10 g/L. Thereafter, bacterial viability was determined by measuring supernatant fluorescence at ex. 540 nm and em. 590 nm with a Chameleon V plate reader (Hidex, Turku, Finland). To quantify the effective viability, first the same experiment was performed with different known concentrations of freely suspended bacteria and calibration curves were obtained. Thereafter, we calculated the fluorescent signal for the corresponding initial bacterial concentration of the particular gel-cast or 3D printed sample. Lastly, we quantified the effective viability by dividing the values of fluorescent signal obtained from the immobilized bacteria test by the corresponding values obtained from the freely suspended bacteria tests.

To confirm the results for immobilized bacterial viability using the resazurin-based assay, a similar test was performed with the WST-1 assay. This assay uses a colorimetric method to define cell viability which is based on the conversion of the tetrazolium salt WST-1 (light pink color) into soluble formazan (orange color) by viable cells. After 24 h genipin crosslinking, three replicates of both gel-cast and 3D printed samples with 0% and 30% alginate containing *E. coli* were incubated for further 1 h, at 37 °C and 160 rpm in a solution of PBS with 10% of WST-1 stock solution. Thereafter, absorbance measurements of the supernatant were performed at 450 nm with Chameleon V plate reader. A calibration curve was obtained from testing different known concentrations of freely suspended bacteria. Furthermore, a control test of nanocomposites without bacteria was performed to assess the influence of the material on both measurements (resazurin and WST-I assay).

## Results and discussion

### Chitosan crosslinking

**Fig. 2:**
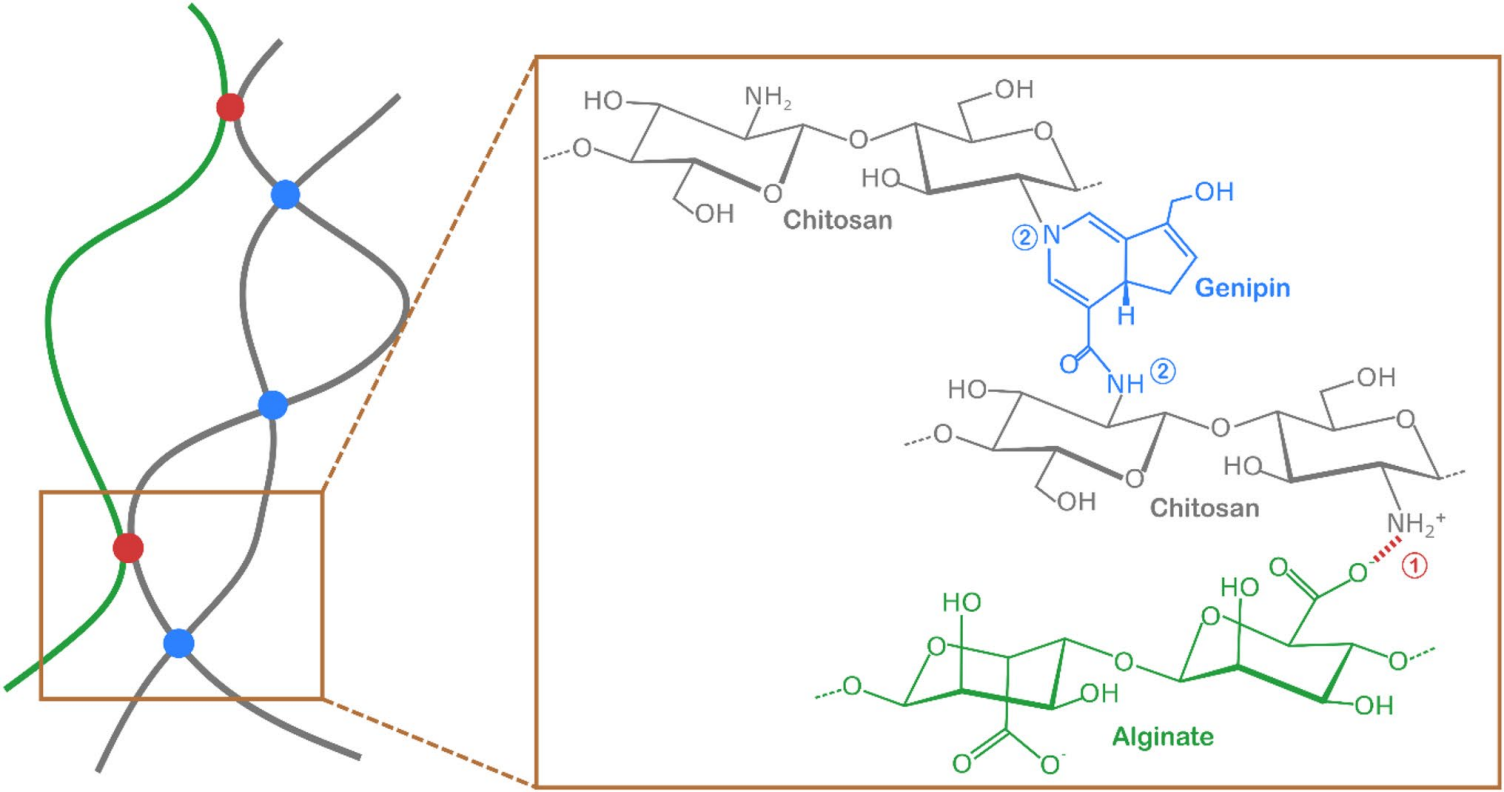
Illustration of the dual-crosslinking of chitosan: (1) electrostatic interaction between the carboxylic acid groups of alginate and amine groups of chitosan; (2) covalent bond formed between the amine groups of chitosan and genipin

Bioinks must accomplish several requirements for processing with a 3D bioprinter. The main challenge here is to develop a feedstock that meets both printability and biocompatibility requirements and fulfills the criteria dictated by their application. In the case of bioprocessing, the main criteria would be a material which does not dissolve or lose its mechanical properties during extended periods of time in the processing environment. Here, we develop a chitosan/alginate/alumina nanocomposite gel for bacteria encapsulation and demonstrate its suitability for 3D printing. The stability of this gel is reinforced by two different crosslinking methods: during bioink preparation, alginate electrostatically interacts with chitosan, and after shaping the gel is interconnected by covalent crosslinking of the chitosan chains with genipin. To this end, first a viscous chitosan gel is prepared in which a high concentration of alumina nanoparticles is suspended. This highly filled nanocomposite gel is then further mixed with alginate leading to electrostatic crosslinking between the amine groups of the chitosan backbone and the carboxylic groups of the alginate molecules (Fig. 2-1). Since electrostatic crosslinking cannot ensure stability of the gels in different media with varying salinity and pH, we utilize a second crosslinking method by the addition of genipin into the feedstock. Note that genipin is added to the feedstock directly before printing, but it only becomes effective several hours after printing due to the slow gelation time of this reaction. Genipin crosslinks the amine groups of the chitosan backbone by a covalent reaction in addition to the electrostatic interactions between alginate and chitosan (Fig. 2-2).

### Rheological characterization

**Fig. 3:**
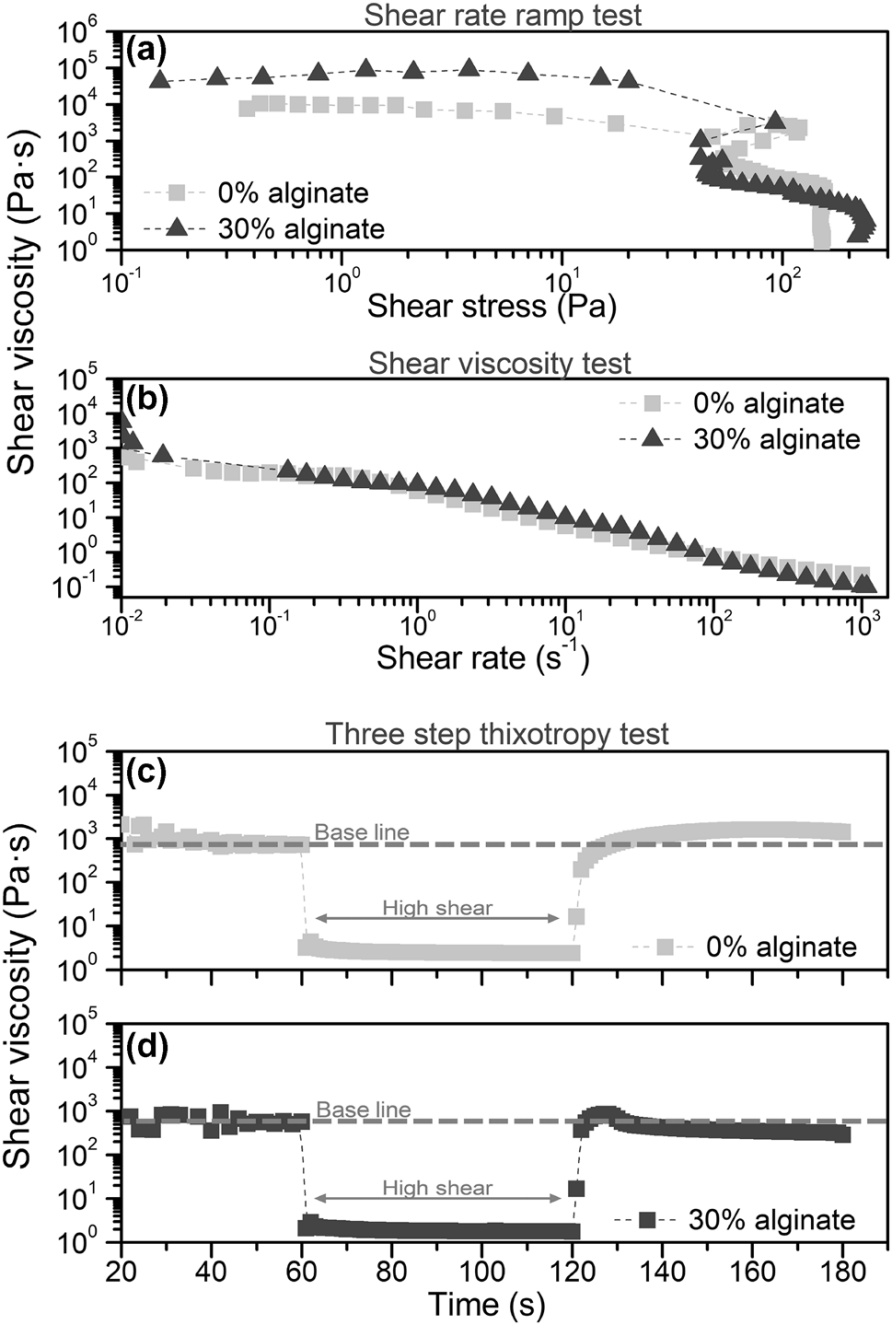
Graph **a** shows the results of a shear rate ramp test plotted to determine the yield point, **b** stepped shear rate test, and **c**, **d** three-step thixotropy test of chitosan/alumina feedstock without (0%) and with alginate (30%)

The rheological properties are crucial for controlling printability and shape fidelity of the bioink and the printed constructs. Therefore, we characterized the rheological properties of the feedstock with a variety of tests that provide information about its behavior before, during, and after the printing process. Specifically, we performed a shear rate ramp to observe the material’s yield stress for flow initiation, a shear rate test to observe the flow behavior, and a three-step thixotropy test to observe the recovery of the feedstock gel after printing. In these tests, we compared the effect of the addition of alginate to the chitosan/alumina mixture. Chitosan and alumina on their own show low viscosities with slightly shear thinning and insignificant yield points which are unsuited for printing (data not shown). A shear rate ramp test was performed to analyze the stress necessary to initiate material flow (Fig. 3a). Both bioinks showed similar curves and a yield point at approx. 120 Pa. This high yield point assures that the material only starts to flow after a suitable stress is applied. The results of the shear rate tests are shown in Fig. 3b. Both gels show shear thinning behavior as a decrease in viscosity with increasing shear rate is observed. A shear thinning behavior is a desirable characteristic for printing since it assures lower viscosity at high shear rate, which facilitates the extrusion process through the nozzle of the printing head. Furthermore, the samples with and without alginate both show similar flow behavior. In comparison, Hafezi et al. [50] printed chitosan-PEG-genipin and obtained similar viscosity results after the initiation of genipin crosslinking, which resulted in a more precise shape of the printed constructs and better mechanical properties.

Post-printing recovery was approximated by a three-step thixotropy test by applying low (0.05 s^−1^), high (50 s^−1^), and low (0.05 s^−1^) shear rate, each for a duration of 60 s (Fig. 3c, d). Both feedstocks, with and without alginate, showed an initial viscosity of approx. 600 Pa·s at 0.05 s^−1^ (base line). Thereafter, the shear rate is increased to 50 s^−1^ and the viscosity of both gels dropped to approx. 2 Pa·s. Subsequently, a shear rate of 0.05 s^−1^ is applied again and we observed the complete recovery of the initial viscosity. Feedstock without alginate took about 9 s for the viscosity to return to the initial value (base line), while feedstocks with alginate recovered much faster after about 2 s. Additionally, the shape of the recovery curve of both feedstocks differs: without alginate, a progressive increase in viscosity is observed until it reaches a plateau slightly higher than the base line, while the alginate-containing feedstock showed a short overshoot compared to the base line viscosity, followed by a slight decrease starting with the base line viscosity. Although both feedstocks, with and without alginate, showed a fast viscosity recovery period, the interactions between chitosan and alginate allowed an almost immediate recovery of the suspensions, which might be a critical factor in printability as discussed below.

### Viscoelastic behavior

**Fig. 4:**
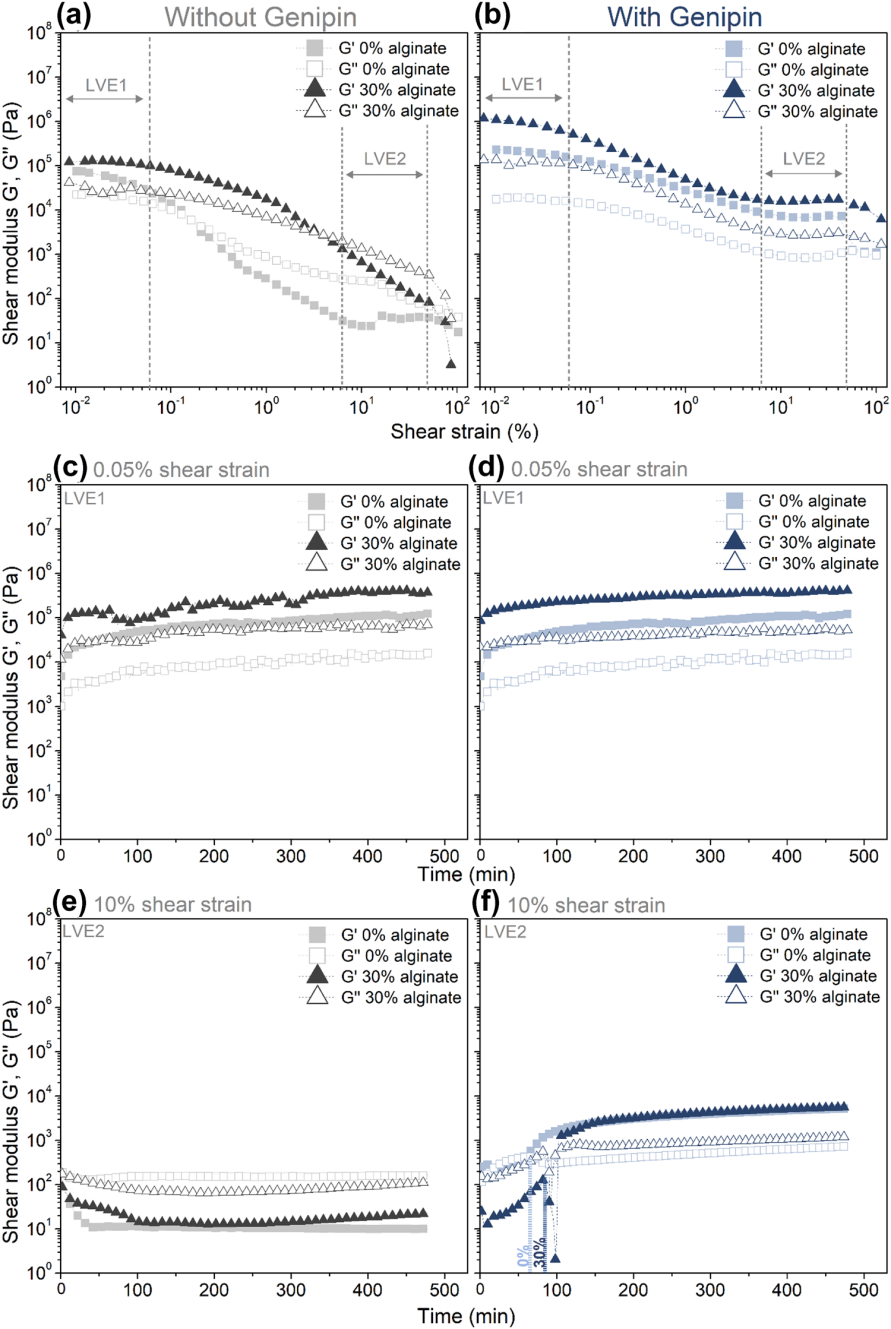
Influence of genipin on the rheological properties of chitosan/alumina-based gels and analysis of the gelation point. The left column (**a**, **c**, **e**) shows the graphs from feedstock without genipin and the right column (**b**, **d**, **f**) displays the effect of the addition of genipin. In the amplitude sweep of the crosslinked suspension (**b**) two LVE plateaus LVE 1 and LVE 2 were observed. Based on these two LVE regions, time tests were performed with the amplitude of LVE1 (**c**, **e**) and LVE2 (**d**, **f**) for suspension with and without genipin. All experiments were performed without (0% alginate) or with alginate (30% alginate)

Oscillatory rheological tests are used to characterize viscoelastic materials by measuring the elastic (*G*´) and the viscous (*G*´´) modulus. First, oscillatory deformation amplitude (strain) sweeps were performed in both non-crosslinked (Fig. 4a) and genipin-crosslinked (Fig. 4b) gels with and without alginate. At low deformation amplitude, all samples showed solid-like behavior with *G*´ higher than *G*´´. This is already evident in the other gel-like properties discussed above, like high viscosity and a pronounced yield point. For samples without genipin or alginate this solid-like behavior was observed up to 0.2% shear strain. Afterward, *G*´´ is higher than *G*´, which manifests in a fluid-like behavior. Adding alginate to the chitosan/alumina feedstock extended the solid-like behavior up to 4% shear strain. Apparently, the additional electrostatic interactions introduced with alginate enhance the polymer network flexibility from the feedstock without increasing the overall viscosity (compare Fig. 3). Furthermore, both viscoelastic moduli of the feedstocks containing alginate are higher than without alginate over the whole deformation range confirming again the influence of alginate in the polymer network which was not visible in the rotational rheological tests.

Deformation amplitude tests with genipin were carried out after incubation with the gelling agent for 24 h at 37 °C. Solid-like behavior was observed throughout the whole shear deformation range for genipin-crosslinked gels with and without alginate (Fig. 4b). Furthermore, overall higher moduli were observed for gels containing alginate than without it. Additionally, genipin-crosslinked gels showed higher moduli over all shear strains than gels without genipin. Interestingly, with genipin two linear viscoelastic (LVE) regions in the form of plateaus with parallel moduli from 0.01 to 0.07% (LVE 1) and from 5 to 45% (LVE 2) were observed (Fig. 4), while only one initial plateau was observed without genipin. This phenomenon is often related to shear banding which might occur in complex fluids that support two different states of apparent viscosity for either the same shear rate or shear stress [26]. In this case, the occurrence of two LVE regions is most likely caused by the activation of the two different types of networks present in the material.

To further investigate this phenomenon, we performed time tests with constant shear strains well within either of the LVE regions (0.05% for LVE 1 and 10% for LVE 2), in both genipin-crosslinked and non-crosslinked suspensions. Without genipin, time tests performed with and without alginate with 0.05% shear strain (Fig. 4c) showed gel-like behavior over the whole duration of the measurement with a slight increase of *G*´ and *G*´´ over time. As before, the addition of alginate generally increases the moduli. Conversely, with shear strain of 10% both gels show liquid-like behavior with *G*´´ higher than *G*´ and a decrease in both moduli observed (Fig. 4e).

With genipin, the time tests were performed directly with the addition of the crosslinking agent (no 24 h waiting time as in the strain tests) to allow the observation of the gelation point. At 0.05% shear strain (Fig. 4d), no gel point could be observed and the gels behave almost exactly like the gels without genipin crosslinking (Fig. 4c). At 10% shear strain (Fig. 4f) liquid-like behavior is initially observed for both feedstock compositions due to deformation beyond the first LVE region as without genipin. However, after approx. 60 min, a sol–gel crossover point is observed for samples without alginate after which the sample regains gel-like behavior. Thereafter, the moduli continue to increase slightly during the observed time frame due to continued crosslinking. For samples containing alginate, a disruption of the curve is observed after around 80 min and after 100 min the sample regains a gel-like behavior. This reproducible disruption might be caused by slipping or the intermittent formation of shearing bands, originating from the high shear strain. Afterward, the network was reestablished due to further crosslinking and the moduli continuously increase reaching values similar to those without alginate. Accordingly, the presence of alginate seems to play a minor role after the sol–gel point, which is expected, since the network is now dominated by the covalent genipin crosslinking and the contributions from the electrostatic interactions between alginate and chitosan/alumina are broken up due to the high shear strain.

Accordingly, these sets of rheological experiments unequivocally characterize both networking mechanisms: the fast and weak network formation in the particle-filled chitosan gel which is enhanced by electrostatic interactions between the negatively charged alginate and the positively charged chitosan and alumina which are apparent at LVE1 and the slower but stronger and more flexible covalent crosslinking between genipin and chitosan at LVE2. This also shows that only the chitosan/alginate/alumina gel determines the printability of the feedstock, while the genipin crosslinking slowly reinforces the structures and ensures long-term stability.

### Printability characterization

Next to the rheological behavior, several other parameters can influence shape fidelity and integrity of printed filaments, such as bioink homogeneity and crosslinking. Feedstock printability (Pr) was assessed by measuring the spacing between printed filaments of a grid structure. Ideal feedstocks for bioink (Pr = 1) should demonstrate a clear morphology with smooth surfaces and constant diameters of the extruded filament, which would result in regular grids and square holes in the fabricated constructs. If the feedstock shows overly solid-like behavior, an irregular spacing (Pr > 1) would be observed, while a more liquid-like feedstock would result in a pronounced circular spacing (Pr < 1) due to filament merging.

**Fig. 5:**
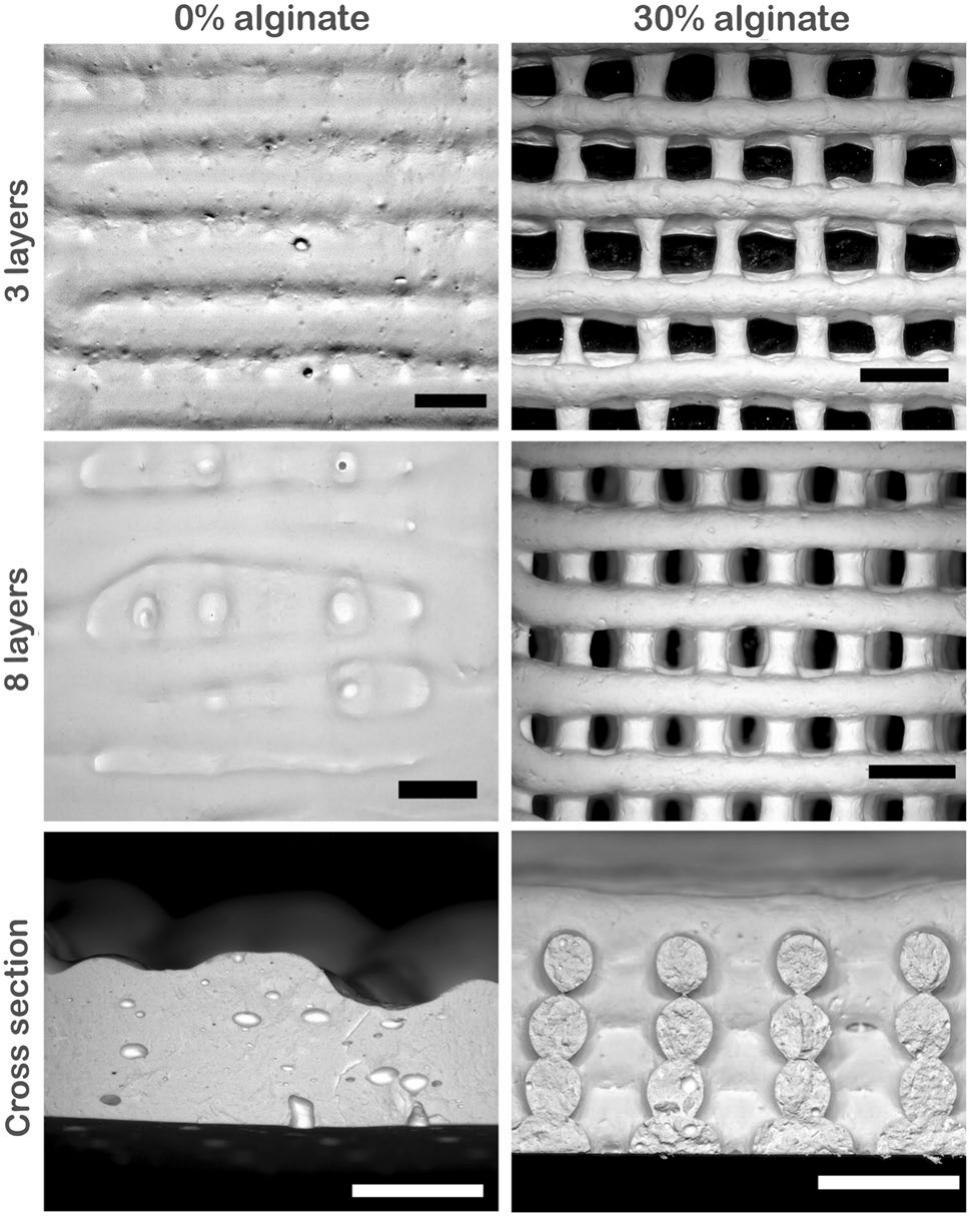
3D printed nanocomposite gels after 3 and 8 printed layers viewed horizontally from above as well as toward a vertical cross Sect. (8 layers) from samples without (0%) and with alginate (30%) (scale bar: 2 mm)

Since genipin does not play a role in the immediate printability, chitosan gel-suspensions without covalent crosslinking and with and without alginate were printed through a ø 0.93 mm nozzle into a lattice cuboid (final size 2 × 2 × 1 cm) and the printability was analyzed after printing 3 and 8 layers (Fig. 5). The printed chitosan/alumina constructs without alginate viewed horizontally from above or looking at a vertical cross section showed a smooth surface of the printed filaments but no spacing could be observed after either 3 or 8 printed layers. Conversely, when the chitosan/alumina feedstock is printed with alginate, viewed horizontally, square shapes between the printed channels were observed after printing 3 and 8 layers, with Pr values of 1.07 ± 0.1 and 0.96 ± 0.04, respectively. Furthermore, although the layers merged to some degree, resulting in stacked filaments, the circular forms of the printed filaments with 796 ± 35 µm diameter could be observed clearly in the vertical cross sections. The differences between both feedstocks’ printability corresponds to their rheological behavior. As discussed above, the rheological behavior with and without alginate showed similar patterns of yield stress and shear rate behavior. However, the much faster recovery time and the overall higher viscoelastic moduli of alginate-containing feedstock contributed to a higher shape fidelity of the printed structures.

Comparison of our results with the study by Heidenreich et al. [59], which analyzed rheological properties of collagen–chitosan bioinks without crosslinking, shows that our chitosan/alumina feedstock has higher viscosity, and the viscoelastic behavior and printability of chitosan/alumina-containing alginate showed better results as well. Nevertheless, the printing tip used in Heidenreich et al. study was half the size of the one used in this paper.

### Material dissolution stability

**Fig. 6:**
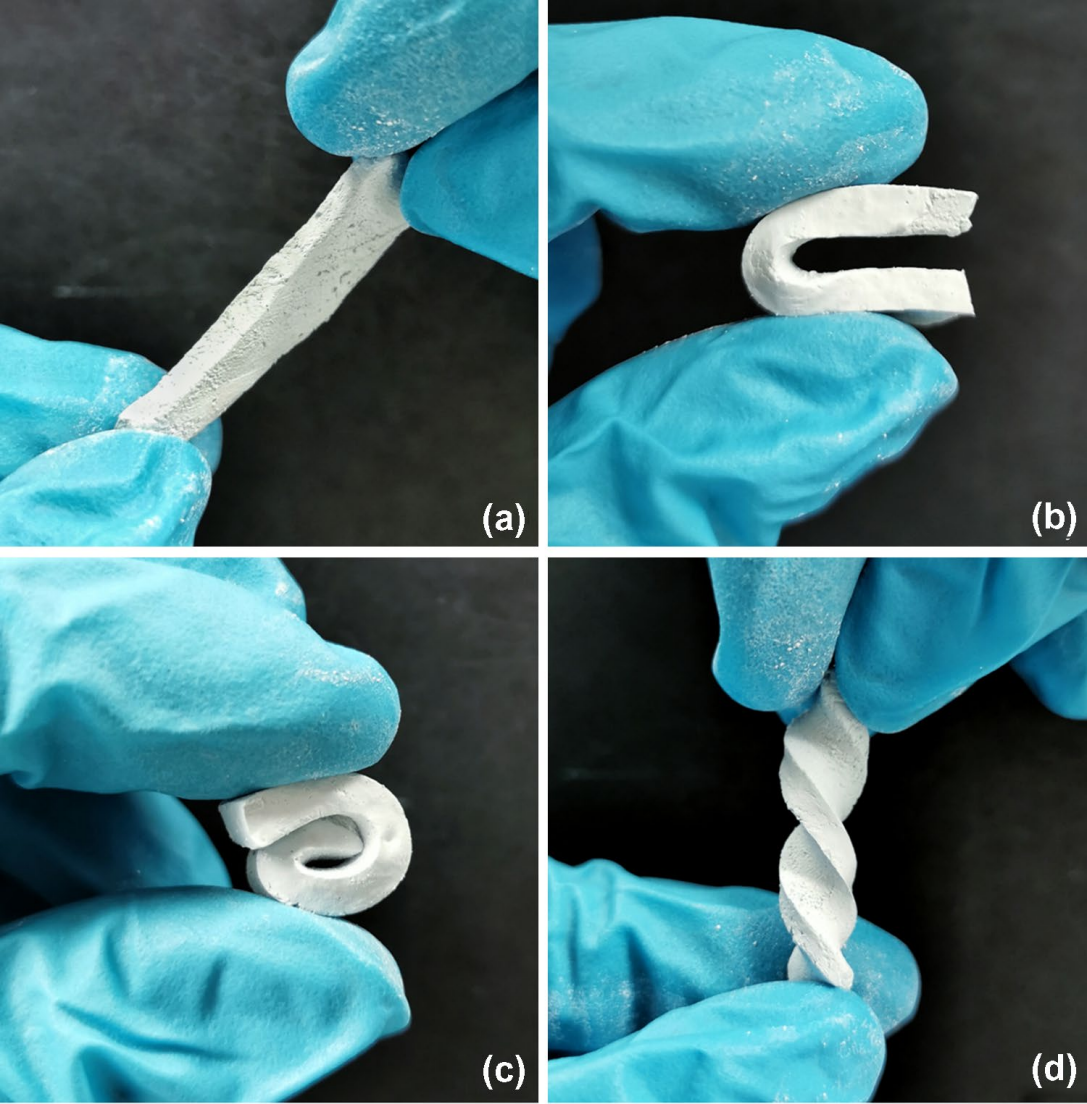
Capacity of deformation of genipin-crosslinked chitosan/alginate/alumina nanocomposites

To visualize the materials’ behavior and their long-term dissolution stability in different media, chitosan/alumina nanocomposite gels with and without alginate and containing genipin were prepared and incubated for crosslinking for 24 h at 37 °C. To visualize material behavior, the gel-cast (non-printed) samples were cut into rectangular pieces (3 × 1 × 0.3 cm) and deformed in different load directions (see Fig. 6 which only shows samples with alginate). It was possible to reversibly deform the nanocomposite material in different directions showing the high elasticity of the material even with the high particle contend. Furthermore, no differences were visually observed between samples with or without alginate.

**Fig. 7:**
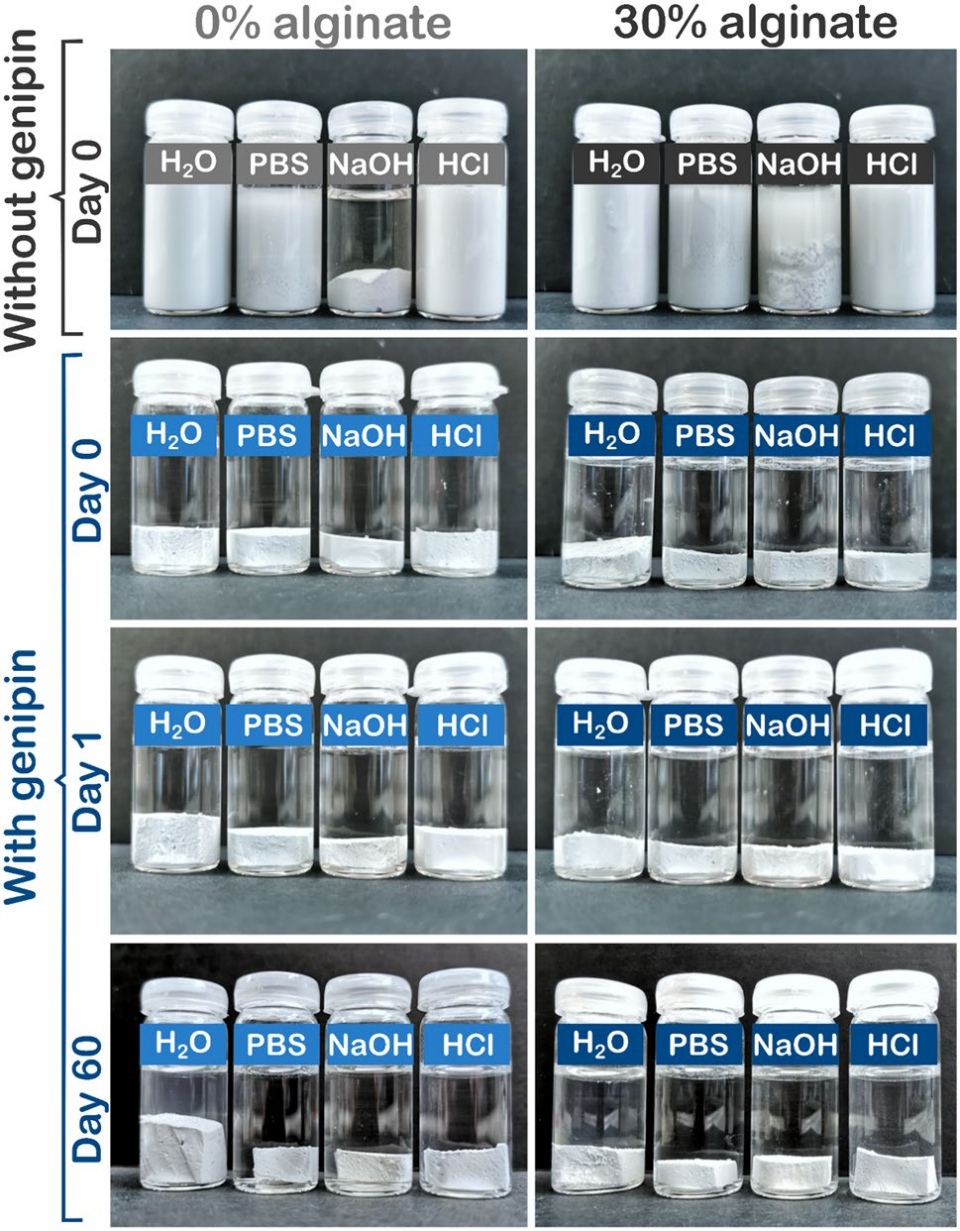
Stability of **a** nanocomposite gels without genipin crosslinking after 24 h and **b**, **c** genipin-crosslinked chitosan composites without (0%) and with alginate (30%) in different media (water, PBS, 1 M NaOH, and 1 M HCl) after 24 h (**b**) and 60 days (**c**)

Long-term stability of the non-crosslinked feedstocks and crosslinked materials against dissolution was assessed by submerging the samples in different media. For that, the crosslinked nanocomposite samples were removed from storage at 37 °C and submerged in H_2_O, PBS, NaOH (1 M), or HCl (1 M), while non-crosslinked feedstocks were directly submerged in each solution and deviations in size or shape were visually analyzed after 60 days (Fig. 7). After day 1, the samples were vigorously shaken for 15 s every four days and also just before imaging. Dissolution of the polymer bonds can be qualitatively visually determined by observing a deviation in shape of the samples as well as an alteration in turbidity of the liquid medium. Change in turbidity by sample dissolution is due to the release of alumina particles from the sample. Complete dissolution was observed after 24 h without genipin crosslinking when samples were submerged in water, PBS, and 1 M HCl, while in 1 M NaOH no dissolution was observed for samples without alginate and a partial dissolution for samples with 30% alginate was observed suggesting insolubility of chitosan in basic pH. Moreover, neither dissolution, swelling, nor shrinkage was observed for genipin-crosslinked gels with and without alginate when these samples were submerged in PBS, 1 M NaOH, or 1 M HCl after 1 and 60 days. However, both crosslinked samples, with and without alginate, did swell in pure water after one day incubation as a consequence of osmotic gradients, although to a lesser degree for samples containing alginate. Most likely, the electrostatic crosslinking of the carboxylic groups of the alginate partially shields the cationic amine groups of the chitosan from contributing to the osmotically induced swelling of these hydrogels.

### Bacteria viability

**Fig. 8:**
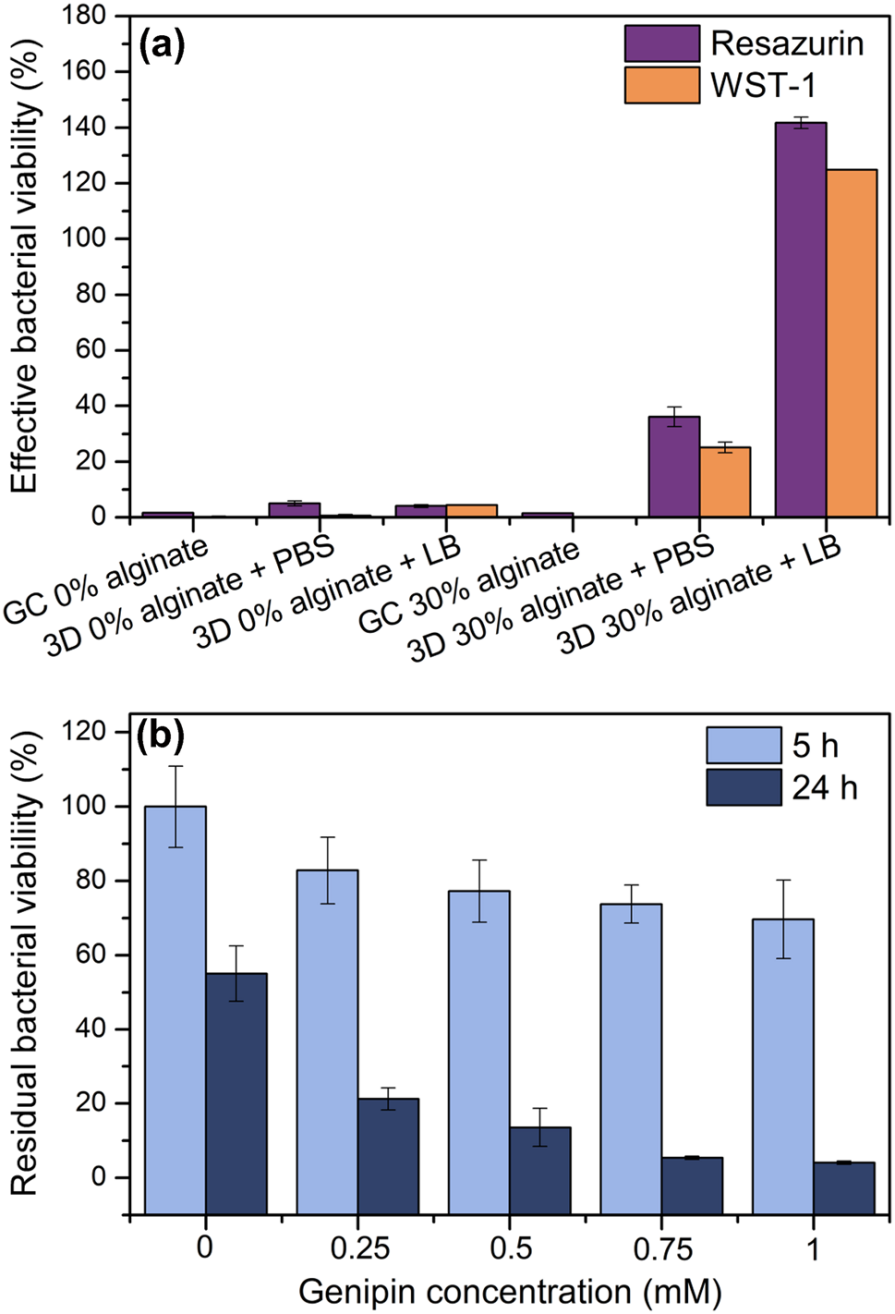
Bacterial viability of embedded *E. coli* in genipin-crosslinked chitosan/alumina composites without (0%) and with alginate (30%) after 24 h genipin crosslinking using resazurin assay and WST-1 assay at 37 °C (**a**). Bacterial viability was measured after two different processing routes: gel casting (GC) and 3D printing (3D), where 3D printed samples were either submerged in PBS or in LB medium during crosslinking to avoid drying. **b** Effect of different concentrations of genipin on the viability of freely suspended bacteria

Lastly, the compatibility of embedded bacteria with the nanocomposite gels was exemplarily characterized for *E. coli* bacteria. Next to the chemical composition of the bioink material, the crosslinking method and the printing process could influence bacterial viability. Thus, to determine the influence of the genipin-crosslinked nanocomposite on bacterial viability, we measured the viability of the immobilized bacteria by cellular reduction of resazurin (blue color) into resorufin (pink and fluorescent color). Measurements with non-crosslinked nanocomposites were not performed due to complete dissolution of the material (Fig. 7a). Genipin was incorporated into the chitosan/alumina gels with and without alginate with a final concentration of 0.20 and 0.26 mM of genipin, respectively, and the feedstock was either poured into a petri dish (gel-cast samples) or printed as described above. Additionally, some of the printed samples were submerged in LB medium instead of in PBS during crosslinking. Bacteria viability was then quantified by the resazurin assay after 24 h of genipin crosslinking (Fig. 8a) and compared to the viability of the same quantity of freely suspended cells. It is important to note that the effective bacterial viability was obtained in these experiments which relates to the viability of the cells accessible by resazurin molecules and the corresponding resorufin metabolite which could release the sample.

Here, the non-printed samples showed almost no effective viability for both gels with and without alginate. Comparably, 3D printed samples without alginate in PBS or with the addition of nutrients (LB medium) also showed no effective viability. Conversely, alginate-containing gels crosslinked in PBS showed approx. 30% effective viability, while samples crosslinked in 6.6% LB solution showed approx. 135% effective viability. The low viability of the bacteria in chitosan/alumina composites without alginate could be a result of the antimicrobial properties of chitosan or of genipin [23] in combination with the poor accessibility of the bacteria inside the nanocomposite structure. Consequently, we analyzed the influence of different genipin concentrations on bacterial viability after 5 and 24 h of incubation (Fig. 8b). Here, genipin shows low influence on bacterial viability after 5 h of incubation but a more pronounced influence can be observed after 24 h. Due to the lack of nutrients, bacterial viability is reduced to 50% after 24 h incubation in pure PBS but genipin-containing suspensions showed a more noticeable reduction of bacterial viability to 25% for 0.25 mM samples and to 7% for 1 mM samples. Furthermore, a slight change of color from colorless to light blue could be observed when the genipin concentration was further increased (data not shown). The blue color is a characteristic of the reaction of genipin with amino groups [23] and a color change of the bacterial solution might indicate that genipin reacted with amino acids of the cell membrane. This shows that genipin in the nanocomposite gels is moderately harmful to bacterial viability which is further substantiated by the absence of an effect of the LB medium which would only enhance proliferation and viability of living and accessible bacteria. Fessel et al. [52] prepared a collagen with tendon cells and incubated the material for 24, 72, and 144 h with supplemented medium and different genipin concentrations up to 20 mM to measure the toxicity of the crosslinker. They also report strong change of color to dark blue with increasing genipin concentration. Furthermore, genipin concentrations higher than 2.5 mM resulted in partial cell death, in which the effect increases with concentration and incubation time. Thus, by tailoring genipin concentration and incubation time the level of toxicity can be controlled.

Likewise, the addition of alginate to gel-cast samples had no effect on the bacterial viability and no viability could be observed. In contrast, for 3D printed samples crosslinked in PBS, 30% bacterial viability could be registered. This difference between printed and non-printed samples is most likely a result of the higher accessible surface area and especially the lower volume to be penetrated by the assay molecules in the printed structure. Furthermore, adding LB medium to the 3D printed samples during crosslinking further increased bacterial viability from 30 to 135%. This increase in viability over 100% indicates that the entrapped *E. coli* were protected by the alginate from genipin and cells were able to proliferate inside the structure. Note that any free bacteria that proliferated outside of the structures were removed before testing by washing with PBS. Additional tests were performed with the WST-1 assay for validation and similar results were obtained (Fig. 8). These results show that tailoring material composition can also mitigate the toxicity of genipin and chitosan on bacteria.

## Conclusion

In conclusion, we developed a new feedstock suitable for 3D bioprinting with embedded bacteria. The feedstock is based on a highly filled chitosan/alginate/alumina nanocomposite with optimized rheological properties regarding shear thinning, high yield stress and fast recovery time. Electrostatic crosslinking of chitosan/alumina and alginate considerably increased shape fidelity after printing, allowing to further reinforce the material by covalent crosslinking between chitosan and genipin. Genipin-crosslinked gels showed two LVE regions which could be related to the different types of networks present in the nanocomposite gels. The first plateau corresponds to the alginate-reinforced chitosan/alumina network, while the second plateau activates the covalently crosslinked chitosan connected by genipin. Genipin-crosslinked chitosan composites could withstand high deformation and showed excellent stability in PBS, NaOH, and HCl solutions. Even though in water genipin-crosslinked composites without alginate showed swelling, this effect could be minimized with alginate-crosslinked chitosan. Additionally, we analyzed the effective viability of *E. coli* embedded inside the nanocomposite materials. Here, we observed no bacterial viability of the samples without alginate in either printed or non-printed state, which might be related to the moderate antibacterial activity of genipin, the reported antibacterial activity of chitosan, and the poor accessibility of the bacteria inside the structures. However, 3D printed alginate-containing composites showed 30% effective viability, while non-printed materials showed again no viability. Accordingly, the printed sample geometry resulted in better accessibility of the embedded bacteria which allowed a higher turnover rate of the assay molecules. Furthermore, the alginate seems to protect the bacteria from the antibacterial activity of genipin and chitosan. Once bacteria were alive and accessible, the effective viability could be further improved from 30 to 135% by incubating the printed samples with LB medium. These results demonstrate that we were able to create a feedstock material for 3D printing with long-term stability against dissolution and in which viable bacteria could be embedded. Such materials pave the way toward innovation in bioprocessing with customized carrier geometries tailored for various microorganisms in a wide range of bioreactor environments.
